# Digital Economy and Environmental Sustainability: Do Information Communication and Technology (ICT) and Economic Complexity Matter?

**DOI:** 10.3390/ijerph191912301

**Published:** 2022-09-28

**Authors:** Asif Khan, Wu Ximei

**Affiliations:** School of Law, Zhengzhou University, Zhengzhou 450001, China

**Keywords:** digital economy, ecological footprint, environmental sustainability, economic complexity, ICT, economic growth, G-seven

## Abstract

In the current era of digital economy, the role of information communication and technology (ICT) and economic complexity are important for controlling environmental unsustainability and formulating policies to deal with ecological concerns. However, the relationship between digital economy and environment has been studied widely; nevertheless, the relationship between ICT-based digital economy, economic complexity, and ecological footprint has not been studied extensively. Therefore, the aim of current study is to fill the existing gap by investigating the relationship between ICT, economic complexity, and ecological footprint in the case of G-seven (digital) economies. Furthermore, the past research studies were usually based on carbon emissions to measure environmental sustainability, while this study fills the gap using ecological footprint as a proxy for environmental degradation. By using the panel data over the period of 2001–2018 for G-seven economies, this study performs first-generation as well as second-generation unit root testing methods. Findings of both Pesaran’s and B&P’s cross-sectional dependence testing approaches confirm the presence of cross-sectional dependence across all G-seven economies. The empirical findings of cointegration (Pedroni and Kao) tests verify a stable long-run association between ecological footprint, ICT import, ICT export, economic complexity, economic growth, and other control grouped variables. The empirical evidence obtained from the fully modified OLS model suggests that ICT export, economic complexity, and economic growth enhance the intensity of ecological footprint, while ICT import, research and development (RD), and trade are helpful in reducing ecological footprint in G-seven economies. These empirical findings obtained are verified by pooled mean group-ARDL (PMG-ARDL) methodologies and confirm that there is no inconsistency in the results. On the basis of these results, some policy implications for ecological footprint, ICT, and economic complexity are discussed.

## 1. Introduction

The challenge of climate changes and environmental degradation are not national issues now, but these are a global challenge and cross the national border [1]. Issues related to climate change have been top priorities of researchers because they are one of the greatest challenges faced by developing as well as developed economies [2]. All developing and developed economies are facing changing weather patterns and sea level, which badly interrupt people’s lives and structure of economies. According to the Inter-governmental Panel of Climate Change (IPCC), the release of greenhouse gas (GHGS) and climate change are increasingly considered to be a serious environmental problem, which has a long-term adverse impact on wellbeing. The increase in greenhouse gas in the atmosphere is the most important determinant of climate change and global warming [3]. As stated by National Aeronautics and Space Administration (NASA), with rise in GHGS emissions (i.e., per capita ecological footprints and carbon emissions), global warming has augmented by 1.6 degree Fahrenheit (°F) since the Industrial Revolution, which is a very alarming situation for life on this planet. The United States Environmental Protection Agency (US-EPA) stated that, since 1990 to 2010, the global emissions increased by 40%, reaching nearly 46 billion tons. These extreme climate changes result in global warming and extreme weather-related events, for instance, heavy rainfall, heatwaves, hot spell, and drought repeatedly seen in the last few years. These climatological changes significantly influence our ecosystem and human lives [4]. To handle these severe issues of climate change and environmental degradation, numerous global agreements and treaties came to pass, such as United Nations Framework Convention on Climate Change—1992 (UNFCCC-1992), Kyoto Protocol-1997, and Paris Climate Agreement—2015 (PCA-2015). These agreements are based on coping with the growth of global warming below 2 degrees centigrade (°C), while, to accomplish this goal, many developed and developing nations have signed to obstruct inefficient energy structure. Nevertheless, regardless of these accords, global warming was on the highest peak and carbon footprints were still growing at the rate of 2.8% in 2018 [1]. Growing carbon footprints compelled the social scientists, researchers, and environment economists to identify the factors influencing ecological unsustainability.

Although the achievement of the highest economic growth is the end goal of any country, developed countries are mostly concerned about their environmental sustainability [5]. In contrast, several less developed economies are ignoring the problem of environmental issues while achieving their desirable target of economic growth. Consequently, the vulnerable and more impoverished people of the society might be suffering more. Hence, international co-operation and global community, in undertaking these indefinite problems, could help developing economies to grow by more maintainable means [6]. Recently, in many studies, the effect of economic growth on carbon emissions has been inspected, while these studies also investigated Environmental Kuznets Curve: Heidari, et al. [7] for ASEAN economies, Malik, et al. [8] for Pakistan, and Khan, Yahong and Chandio [5] for G-seven economies.

The developed economies, such as the G-seven group, led a massive increase in economic growth during the recent decade. As a matter of fact, G-seven economies hold about 60% of global wealth (USD 320,000 billion) (World Bank, 2021). In contrast, developing economies are becoming emerging economies by improving their human capital, energy supply structure, infrastructure, and economic growth. Among them, the economic production and financial output of China and India were about USD 30,000 billion, which were far more than as compared to the United States (USD 19,400 billion) and all European Union (EU) countries (USD 21,000 billion) in 2017–2018 [9]. The enormous economic growth in these countries leads to a massive increase in the demand for energy consumption. As International Energy Agency (IEA) stated, all G-seven economies and a few emerging economies are the biggest carbon emitters. For example, in the list G-seven group, USA is on top (producing 4800 metric ton), and Japan (1100 metric ton), Germany (718.9 metric ton), Canada (548 metric ton), France (306.09 metric ton), UK (359 metric ton), and Italy (321.5 metric ton) are the highest deficit of ecological footprint and largest carbon producer countries (IEA, 2020). Contrary to the background framework of the study, the current research proposes to examine the impact of information and communication technologies (ICT) and economic complexity on ecological footprint in G-seven economies.

In the current time of digitalization, the contributing role of ICT on environmental quality and economic growth has been discussed by numerous environmental economists. In the path of economic growth, ICT contributes to economic growth in real terms in the following three ways. First, the emerging of ICT cuts the total factor costs (TFC) by providing good direction of communication with lower transaction costs, for instance, online business, online banking, online buying and selling, E-commerce, and numerous applications of smartphones [10]. Second, corresponding transforms of finance assist and facilitate stockholders and boost monetary insertion. Third, it changes and improve industrial structure by developing advanced production methods [11]. In contrast, the environmental significances of ICT have been largely considered in the environmental economics literature. Many researchers argued that the development of ICT causes more hazardous construction to our ecosystem. Thus, arguments require more empirical studies on the relationship between ICT and environmental quality. The advance era of ICT began in the early 1990s, which increasingly reshaped all characteristics of human wants and their wellbeing. The ICT gave the impression to humans of an innovative strong point with the actual potential to grow the forthcoming life by transporting society and cultures closer, overcome the restriction between countries, and encourage business activities, for instance, trade openness, economic complexity, and foreign direct investment [12,13,14]. Indisputably, the emergence of advanced ICT systems could bring more aptitude for enhancing per capita income of the people by endorsing new business markets, cumulative revenue, and stabilizing pricing, unemployment, and costs of transaction [15,16].

However, the contributing role of ICT is not limited to real economic growth; the enormous infrastructure development related to ICT and other macro and micro uprising can also control the environmental performance by means of the cost’s upshots, consumption, and their substations. Primarily, technological infrastructure based on ICT improves utilization of energy consumption, while collecting, installation of machineries, and distribution of these machines and equipment generates toxins, which degrade the ecological system [12,15,16,17]. The production and consumptions of ICT mostly depend on unfavorable resources, such as manufacturing of computer system using about one thousand gists, while those manufacturing machineries are harshly unfavorable for our ecosystem; hence, as stated by Global E-sustainability Initiative (GESI), between 2.5% and 3.5% of global carbon emissions are produced by the manufacture of ICT devices [18,19]. In addition, ICT can also control the climatic pathway of substitution effect (SF) and consumption effect (CE), which comprise dematerialization, demobilization, and carbon decarbonization exercises [20]. Nevertheless, the SE reduces the consumptions of nonrenewable energy and, therefore, pollution level by substituting the conservative and conventional commodity to effective commodities, particularly in the current new coronavirus 2019 (COVID-19) time [13,21]. For example, the consumption of electronic books (e-books) and emails have reduced the aggregate demand for conventional books and letters. Likewise, virtual and business meetings, online classes (education/schooling), e-banking (online banking), e-shopping, and e-commerce significantly overcome physical appearance and decreased the traveling expenditures [22]. Similarly, the up-to-date development in transport system (GPS system, etc.) has enhanced the traffic flows and sequences by decreasing the overcrowding level that increases carbon emissions [20]. The emergence of ICT has miscellaneous consequences on environmental quality (carbon emissions, ecological footprint, and greenhouse gases). On the one hand, the development of ICT (digital sector) is directly linked to the establishment of advanced, renewable, and cleaner energy and decreases in unsustainable utilization (misuse) of productions units. On the other hand, ICT also leads to modern device and energy structure with lower power dependence, which further leads to diminishing the dependency on nonrenewable energy consumption and fossil fuels, and, therefore, protects environmental sustainability.

In current prevailing economic structure, economic complexity index (ECI) has been considered with widespread attention among many social and environmental scientists, because it is also a most consistent and valid indicator of economic growth, as with ICT [23]. On the one hand, ECI upsurges the range and verity of the products, while accelerating the further production and investment, which increase the energy consumption that leads to an increase in pollution and environmental unsustainability. On the other hand, ECI has more capacity to maintain the sustainability of the environment, as this comprises research and development (R&D), more ICT-related machines and equipment, renewable and cleaner technology, and more environmentally friendly productions [24].

In the context of the above discussed arguments, the current study investigates the impact of information communication and technologies (ICT) and economic complexity (ECI) (along with economic growth, research and development, foreign direct investment, trade ration, and other important control variables) on ecological footprint (EcoFP). To do so, we used fully modified ordinary least square (FM-OLS) and pooled mean group-autoregressive distributive lag (PMG-ARDL) regressions models over the time spanning from 2001 to 2018 for the G-seven group of countries (G-seven: Canada, France, Germany, Italy, Japan, United Kingdom, and United States). This investigated group of countries was originally founded in the early 1970s, while, now, they are economically well-developed and technologically advanced nations. In early 2018, these economies collectively comprised approximately 60% of the global wealth for a total of USD 317,000 billion, while their share of nominal GDP was about 45.8%, purchasing power parity-based GDP approximately 31%, and they comprised 770 million people or 10% of the world’s population [25]. So, it can be reasonably expected to find profound empirical evidence on the theoretical framework of the EKC hypothesis, as well as other important environment influencing factors in these regions.

The G-seven group countries are investigated for a few reasons. First, these economies hold 29.9 percent of global GDP and based on digital economy but, at the same time, they have a huge share of global energy consumption; consequently, G-seven are responsible for environmental unsustainability [5]. Second, G-seven economies are advanced, while they are in a changeover phase of digitalization and knowledge. In addition, G-seven economies are more skilled and knowledge-based economies as compared to other developing or emerging economies. Third, the effect of digital economy in terms of ICT and ECI on ecological footprint have been overlooked by many studies in G-seven economies. Therefore, by focusing on these reasons, we have important policy implication for the governments of G-seven economies.

On the basis of the above-mentioned discussion, the current study examines how digital economy, in terms of ICT, affects environmental sustainability in terms of EcoFP. Then, in the context of economic complexity (ECI), it makes a great effort to investigate the direct and indirect controlling mechanisms of digital economy on EcoFP. Furthermore, the possible effect of ECI is explored, along with the influencing mechanism of ICT on EcoFp relevant to ECI also beingverified. The results obtained from the current study can serve as a theoretical guide for testing how digital economy in terms of ICT and economic complexity influence the growth of EcoFP. Temporarily, the results also provide empirical support regarding digitalization of economy, a skills- and knowledge-based industrial process, and enhancement of environmental sustainability in many developed and developing economies.

**Figure 1:**
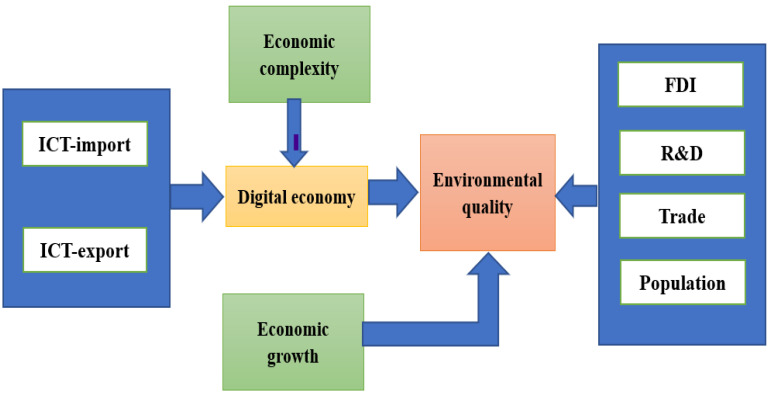
The Conceptual framework of the model. Source: authors’ own derivations.

In this context, we seek to report the following question: what impacts do ICT-based digital economy and economic complexity have on ecological footprint in the case of G-seven economies? We seek to empirically answer the above-mentioned research question by aiming to examine the relationship between ICT, economic complexity, and ecological footprint in G-seven economies by incorporating many control variables. The current research adds to the existing literature in five ways: first, as far as we know, there are hardly any studies that have examined the relationship between ICT-based digital economy, ECI, and EcoFP for G-seven economies. Second, as compared to other papers in the existing literature, in this study, we inspect how ICT (two types of ICTs: ICT-exported goods and ICT-imported goods) and economic complexity (based on countries’ industrial structure and knowledge-based production structure) influence EcoFP (based on carbon uptake land, grazing land, cropland, forest land, fishing ground, and built-up land). Third, besides the wide-ranging proxies of environmental degradation (EcoFP) and digitalization of economy (ICTs and ECI), we have incorporated economic growth (GDP), research and development (R&D), foreign direct investment (FDI), trade ratio, and population as important control variables to avoid biasness. (Figure 1 describes the conceptual framework of the model.) Fourth, regarding the econometric models, the current study used FM-OLS and pooled mean group-autoregressive distributive lag (PMG-ARDL) regression models to obtain the robustness and validity of the results. Fifth, after obtaining the empirical findings, we suggest a few productive and important policy recommendations for policymakers that might contribute to achieving the target of SDGs.

The remaining structure of the paper is as follows: Section 2 outline the literature review. Section 3 presents data and model specification, while Section 4 discusses empirical results obtained from specified models. Section 5 provides discussions of the study and policy recommendation.

## 2. Review of the Literature

In the existing environmental economics literature, there have been numerous empirical studies related to environmental Kuznets curve (EKC) since the early work of Grossman and Krueger [26], where this model has been verified in many developing, as well as in developed, countries through applying miscellaneous econometric methods and techniques. The relationship between economic growth and environment has been investigated by applying times-series and panel data with other important control variables that contain international trade/trade openness [25], foreign direct investment [27], economic development and tourism management [28], demand and price of energy (renewable and nonrenewable energy) [29], innovation and renewable energy [30], ICT and real income [31], ICT and human capital [32,33], and ICT and innovation [34]. While many empirical studies incorporated ecological pressure measures, such as biodiversity and deforestation, the results of some of these studies were based on carbon emissions to assess the actual quality of the environment. Nevertheless, the carbon dioxide emissions might not be a good measure for the exploitation of our environmental health and its impact, while concentrating only on greenhouse gas emissions decreases the validity of findings.

In the recent existing literature of environmental economics, some empirical studies have utilized EcoFP as a measure of environmental sustainability. EcoFP supports us to recognize well both direct and indirect effects of consumptions and production on the environment [1,5,35]. After the empirical work of Rees and Wackernagel (1996), EcoFP (as a measure of environmental quality) has been utilized in many empirical papers related to environmental economics. This measure of environmental sustainability has become novel scientific evidence of ecology while it measures the quality of environment in the framework of six productive surface areas’ components [36]. Few empirical studies examine the long-run association between EcoFP and economic growth for different countries. Economic growth affected environmental sustainability through a few technical channels; for instance, Baloch, et al. [37] revealed that real income growth within the scale–effect (S-E) deteriorates environmental sustainability, changing technological structure and compromising economy structure. Secondly, the composition–effect (C-E) reduces the detrimental effects of real income growth via economic structure change. Lastly, the techniques–effect (T-E) increases environmental sustainability due to environmentally friendly technologies and implementation of mitigation policy [38]. The dominant role of C-E and T-E over S-E leads to the establishment of an inverted-U or EKC hypothesis between economic growth and environmental quality. This phenomenon is examined again and again; while some studies tested and verified the establishment of EKC hypothesis: Khan, Yahong and Chandio [5] for G-seven economies, Baloch and Wang [39] for BRICS countries, and Huang, Haseeb, Usman and Ozturk [36] for E-seven and G-seven groups of countries, the empirical work of Amri, et al. [40] in the context of Tunisian economy and Alshehry and Belloumi [41] for Saudi Arabia failed to confirm the EKC hypothesis between economic growth and environment.

However, in this era of digitalization, the significance of ICT and ECI on development has been broadly deliberated in the last few years. According to Schumpeter [42] and the initial definition, the process of industrialization will substitute the low and incompetent economic sectors (private or business sector) with an advanced and modern sector. Therefore, the emergence of ICT and ECI contributes positively in the process of sustainable economic growth and development. These two important measures play a positive role in the development of economies in three main ways. (i) They minimize the production costs via providing low or no costs of transaction and a better system for communication (e.g., online buying and selling or E-commerce); Ozcan and Apergis [43]. (ii) They expand the business sector (both agriculture sector and industrial sector) with more skillful and knowledgeable production. (iii) ICT and ECI transformed all resources, especially financial resources (such as irrevocable guarantees, irrevocable lines of credit, and liquid assets), to more active stakeholders and encouraged financial inclusion [44]. Nevertheless, the impact of ICT and ECI on the environment has been broadly argued in the existing literature of environmental economics. However, some studies stated that the emergence of ICT and ECI is more detrimental than other machines and equipment. Therefore, this leads us to further analyze the relationship between ICT, ECI, and EcoFP.

Hypothetically, one school of thought has appeared as a basis to assume the linkage between digital economy (adaptation of ICT) and environmental sustainability. One is that the modernizations of an ecological system which supports that digital economy in terms of ICT can enhance economic, structural transformation, improved technologies, changes in the industrial process, and, therefore, environmental regulations and mitigation. The emergence of ICT will eventually improve environmental quality and sustainability in terms of low levels of carbon emissions and ecological footprint [45,46]. Another school of thought argues that digital economy in terms of ICT adaptation is basically intended to be heavily dependent on energy consumption (electricity). Therefore, the adaptation of digitalization infers high demand for energy consumption, which leads to environmental unsustainability. This theoretical argument could be justified if the energy is generated from a nonrenewable source [19,47]. Meanwhile, another school of thought that most believe is that the above-mentioned schools of thought hold true arguments and, therefore, ICT will expect to have a neutral effect of environmental sustainability. According to this third school of thought, the competence of low consumption of energy resulting from ICT, which leads us to increase the demand for energy consumption, consequently cancels out any positive impact on environmental quality [48].

The emergence and advancement of ICT progress have different effects on climate change and our ecosystem. It is associated with the formation of environmentally friendly goods and services and consumption of cleaner energy. Furthermore, technological advancement is opening up new apparatus with low nonrenewable energy consumptions and power supply units that are based on renewable energy consumption and, therefore, mitigate environmental pollution. The association between ICT and EcoFP can be studied from different viewpoints: real income [31], human capital [32,49], and economic development [36]. In contrast, it can also be known as a propellent for environmental unsustainability, economic growth, and industrialization progress. Nevertheless, the empirical work of a few researchers revealed that the relationship between ICT and environmental pollution is significantly positive compared to that of financial development in the context of highly developed economies [50].

Apart from ICT, measure of economic complexity, ECI, is positively related with environmental degradation (i.e., ecological footprint) [5]. According to the definition of Hidalgo and Hausmann [51], ECI is a country’s economic structure changes, technological rigorous export, and skills- and knowledge-based production structure in the direction of particular energy utilization pattern. Undeniably, ECI through the skills and education sector plays a very vital role to encourage business activities and improve the mitigation process. For example, Dinda [52] for United States; Huang, Haseeb, Usman and Ozturk [36] for E-seven and G-seven economies; Jin, et al. [53] for Chinese economy; Mensah, et al. [54] for OECD group of countries; and Santra [55] for BRICS countries reported that ECI has a negative relationship with environmental unsustainability. The work of Doğan, et al. [56] examined the dynamic relationship between ECI and ecological pollution for lower-, middle-, and high-income countries. The empirical findings of the study suggested that ECI and environmental unsustainability have a positive relationship in lower- and middle-income economies, while economic complexity increases environmental sustainability in higher-income countries. Similarly, Chu [57] examined the relationship between ECI and environmental pollution by using panel data of 118 economies. The findings tested and confirmed the EKC hypothesis the in investigated region. Using the panel data with different econometric approaches, Dong, et al. [58] evaluated the emission mitigation policy force on China. Likewise, the results revealed that emissions mitigation SDGs-13 decreases diversification in Chinese economy. Moreover, Romero and Gramkow [59] used 67 countries’ data to investigate the association between greenhouse gases emissions and ECI by using different panel techniques. The empirical results suggest that ECI decreases the level of greenhouse gases and also suggested that advanced and complicated production level has to impend to overthrow environmental pollution. In addition, Pata [60] and Shahzad, Fareed, Shahzad and Shahzad [9] scrutinized the dynamic effect of ECI and demand for energy consumption on EcoFP for USA and revealed that increasing demand for energy consumption was positively associated with the EcoFP. Moreover, Neagu and Teodoru [24] stated that the impact of ECI on ecological degradation is more than that of internal and external trade, which ultimately supports achieving the UN target of SDGs, and the role of ECI and consumption demand for renewable energy reduces greenhouse gases in the context of developed economies. The empirical findings further revealed that ECI mitigates ecological unsustainability, even in the long-run.

### Hypothesis Formulation

The role of digital economy (ICTs and economic complexity) is not only restricted GDP growth (economic); the enormous development of ICT-related economic structure and related knowledge- and skill-based industrial structure can also influence the environment by means of their consumption pattern, cost, and substitution effect. At the initial stage, ICT-based technology, and ECI-based machines and equipment increase the demand for energy consumption [21]. Particularly, the installation of ICT-based machines and equipment, accumulation, and their distribution release toxins and waste, which depreciate the sustainability of the environment [17,61]. The production of ICT-related goods and services rely on a huge number of detrimental materials, such as computer systems and other materials being severely harmful for the environment. Moreover, according to reports, more than 2 and less than 4 precent of global emissions are caused by ICT-related productions [36]. On the other hand, economic complexity in terms of transformation of economy increases, the diversity of the knowledge- and skill-based product accelerates, while the extra production and industrial investment needs the demand for energy consumption that increases toxins and waste, and, therefore, environmental unsustainability. Based on the above arguments and the empirical and theoretical studies (mentioned in Appendix A: Literature review’s summary in tabulated form), it can be hypothesized that:

**Hypothesis** **1.** *Information communication and technology impacts on ecological footprint*.

**Hypothesis** **2.** *Economic complexity has an impact on ecological footprint.*

Furthermore, from the above-mentioned discussion and related literatures, we observed that ICT and ECI both have the ability that can increase the quality of environment or increase environmental unsustainability, depending on their level of diffusion in an economy, time period of analysis, and data and method that are incorporated. To the best of our knowledge, only a small amount of empirical research scrutinized the impact of ICT and ECI on EcoFP in the context of developed countries, especially G-seven economies. To the possible extent, the existing literature has overlooked some important environment-influencing factors, such as research and development (RD), that might be some foundation of misspecification and biasness. In addition, to our knowledge, the current research is the first study of its kind that examines the linkages between ICT, ECI, and environmental degradation in G-seven economies over the period of 2001–2018. Our study aims to fill the existing research gap in the literature. The empirical findings obtained from the analysis of the study would deliver initial caveats for policymakers of the targeted region.

## 3. Materials and Methods

We targeted G-seven economies (developed) over the period of 2001 to 2018 (18 years). According to Global Footprint Network (GFN), these countries are in the list of top 10 countries with high EcoFP (GFN, 2018). The data for EcoFP are obtained from the official website of GFN (2021) database. As a dependent variable, per capita EcoFP was used as a proxy for environmental sustainability. Regrading independent variables of our models, we used as many as possible, as well as control variables (see Table 1). We used two types of ICTs (ICT_exp_ and ICT_imp_) as proxies for digital economy; ICP_exp_ measures the percentage of total ICT-related exported goods, while ICT_imp_ measures the percentage of total ICT-related imported goods, ECI presents a country’s composition expression of the production process by inclusion of the knowledge on their diversity (goods exports) and economic growth measure as per capita GDP (USD). Likewise, R&D measures the expenditure on research and development programs (% of GDP), and FDI represents the net inflow of foreign direct investment to the investigated region. Similarly, access to clean energy and technology is the percentage of total population, trade ratio is taken in the share of GDP, and population is taken as how many people living per square kilometer of land area.

### 3.1. Econometric Method and Process

The current study analyzes the relationship between ICT, ECI, and EcoFP, i.e., whether and how ICT reduces ecological pollution in G-seven countries and what is the possible role of economic complexity in this process. In addition, this study also used some control group factors so that it would not omit any important factor. Moreover, all possible variables used in the regressions are converted to logarithmic form to verify homogeneousness in our series. Following the study objective, our EcoFP was model as follows:

(1) $$y_{it}=\beta_{0}+X_{it}\beta_{1it}+{έ}_{it}$$

In the above equation, *y* represents dependent and *X* represents independent variables (including main and control variables). In addition, ‘*i*’ and ‘*t*’ denote cross-sections (countries) and time (years), correspondingly, while *έ* represents stochastic error term.

By considering these variable, our study further proceeds for the specification of the model following Khan et al. (2022), and we present the specification of the model with log relationship as follows (see Equation (2)):

(2) $$ln{\mathrm{EcoFP}}_{it}=B_{0}+B_{1}ln{{\mathrm{ICT}}^{exp}}_{it}+B_{2}ln{{\mathrm{ICT}}^{imp}}_{it}+B_{3}ln{\mathrm{ECI}}_{it}+B_{4}ln{\mathrm{GDP}}_{it}+B_{n}lnX_{it}+{έ}_{it}$$

Since all variables (dependent and independent variables) in Equation (2) are presented in log form, the estimated parameters in log form represent long-run responsiveness (long-run elasticities).

Regarding the estimation process, firstly, the current study investigates the under-examined panel data of G-seven economies for the cross-sectional dependency, which is a very common matter in panel data. To this end, in this study, we have applied Pesaran, et al. [62] CD test and Breusch and Pagan [63] dependency tests. Additionally, the panel unit root (stationarity tests) has been estimated for level I (0), as well as for 1st difference I(1). Given the presence of cross-sectional dependency in a series, we used second-generation stationarity tests. The generalized equational form of first-generation unit root test is presented in Equation (3) as follows:

Δ*Y_it_* = ρ*_iYt_*_−1_ + *ξ_it_λ* + e*_it_* (3)

*Y_it_* = (1 − ρ*_i_*)μ*_i_* +δ*_i_Y_it_*_−1_ + *ε_it_* (4)

In Equations (3) and (4), *i* is different cross-sections (countries), *t* is specific time periods (years), *ξ_it_* and μ*_i_* represent the deterministic component, while e*_it_* expresses the process of stationary series. Regarding second-generation (CIPS) panel unit root testing approach, this paper also suggests a simple stationary test in the occurrence of cross-sectional dependency.

### 3.2. Panel Cointegration Approaches

The existing of cointegrational association among variables can be explored by using Kao panel cointegration and Engle–Granger-based Pedroni test. Based on these tests, the long-run relationship confirms the existence of cointegration; therefore, we applied FM-OLS techniques. Equation (6) provides the general form of the model:

(5) $${Ǎ}_{\text{FM-OLS}}{\left(\sum_{i=1}^{N}{й}_{22i}^{-1}\sum_{t=1}^{T}{\left(x_{it}-{\bar{x}}_{i}\right)}^{2}\right)}^{-1}\;\sum_{i=1}^{N}{й}_{11i}^{-1}{й}_{22i}^{-1}\left(\sum_{t=1}^{N}(x_{it}-{\bar{x}}_{i}\right)y^{*}-T{\hat{E}}_{i}$$

In the above equation:

(6) $$Y*=(y_{\mathrm{it}}-\bar{y})-\left(\frac{{й}_{21i}}{{й}_{22i}}\right)\Delta x_{it}+\left(\frac{{й}_{21i}-{й}_{221}}{{й}_{22i}}\right)\beta \left(x_{it}-{\bar{x}}_{i}\right)$$

(7) $${\hat{E}}_{i}≅\;{Ѓ}_{21}+\Omega_{21i}^{0}-\left(\frac{{й}_{21i}}{{й}_{22i}}\right)\;({Ѓ}_{21}\;+\;{\;\Omega }_{21i}^{0})$$

Thus, *y_it_* and *x_it_* represent dependent and independent variables; moreover, with partition covariance, й is the triangle of lower-case matrix. Similarly, asymptotic covariance matrix for cointegration analysis is denoted by Ω, while Ѓ presents dynamic covariance in the above equations. Furthermore, in the analysis of current study, we also developed the pooled mean group-ARDL (PMG-ARDL) model that allows for the estimation of both short- and long-run coefficients. In the existing literature, many studies suggest several advantages of PMG-ARDL model over FM-OLS model that can be traced by [64,65,66].

After detection of the cointegration between the dependent and independent variables, the next phase is to estimate the degree of the long-run elasticities of the variables used in the model. To this end, we use FM-OLS methodology to calculate the long-run coefficients. The FM-OLS methodology has the capability to tackle the issue of serial correlation and the problem of endogeneity in the analysis of panel data. FM-OLS suggests a nonparametric approach and has to work in a small sample dataset (sometimes called micronumerasticity). Furthermore, we employ another econometric approach to approximate a dynamic nonstationary panel using PMG methodology introduced by Pesaran, et al. [67]. The main advantage of this methodology is that it permits short-run elasticities of the variables for different cross-sections. However, in this study, we only consider the long-run parameter estimates.

## 4. Results and Discussion

### 4.1. Summary Statistic

Table 2 displays descriptive statistics of all possible variables used in regression models. Apparently, dependent and all independent variables provide skewness (positive and negative) and kurtosis; consequently, these skewness and kurtosis deviate us from normal distribution (bell-shaped) and lead us to asymmetric distribution. Moreover, the null hypothesis of JB or Jarque–Bera normality test for mostly all variables except ln ICT_exp_ is rejected, even at 10%, 5%, and 1% levels. Particularly, lnICT_imp_, lnGDPpc, lnECI, lnFDI, lnPOP, lnTRA, lnPOP, lnINF, and lnRD are negatively skewed, while the remaining variables (such as lnEcoFP and lnICT_exp_) obtain positive skewness and, therefore, heavy-tailed distribution.

The phenomenon of heavy-tailed distribution also leads us to apply the most valid and suitable panel techniques to empirically observe the association between ICT_exp_, ICT_imp_, GDP, ECI, and EcoFP (along with other control variables). As discussed above, we use the two latest econometric panel techniques, namely “FM-OLS” and “PMG-ARDL”, to actually reflect the net effect of all independent variables on the dependent variable.

### 4.2. Cross-Sectional Dependence Tests

Similarly, in Table 3, the empirical findings of cross-sectional Pesaran, Schuermann and Weiner [62] and B-P tests suggest statistically significant results of test statistics, which evidence that all cross-sections (countries) are economically and socially independent. The test statistics of both the tests for cross-sectional dependence reject the null hypothesis for all variables used in our regression models.

### 4.3. Panel Unit Root Tests

In order to check the stationarity level and order of integration of all series, we utilized IPS and CIPS panel unit root tests, as shown in Table 4. The IPS is the first-generation stationarity test, while the CIPS is the second-generation test. The empirical results of both the tests for stationarity suggest that all variables seem to have integrated with different order. However, both the tests for stationarity check offer the same findings; the findings of CIPS test for heterogenous panel are very significant compared to other tests because it adopts cross-sectional dependency caused by a single common factor.

### 4.4. Panel Cointegration Tests

On the basis of unit root tests, this study further proceeds to scrutinize the long-run linkages between dependent and independent variables used in our regression models. To this end, two cointegration tests have been applied, namely “Pedroni” and “Kao” cointegration tests. Table 5 suggests that three test statistics (such as Panel v-stat, Panel PP-stat, and Panel ADF-stat) within dimension and two test statistics (such as group PP stat and Group ADF stat) between dimensions are statistically significant under the methodology of Pedroni panel cointegration test. Likewise, the result of Kao panel cointegration test also suggests a statistically significant value under the test statistic of ADF approach. Therefore, the statistically significant results of both the cointegration tests propose the presence of long-run association among the variables, which helps us to employ FM-OLS and AMG-ARDL methodologies.

### 4.5. FM-OLS Panel Model

From Table 6, after verifying the existence of a long-run relationship between the dependent and independent variables, it is, therefore, required to estimates the long-run panel cointegration model. FM-OLS econometric technique is used to assist the aim of the study. In models FM-OLS I to FM-OLS III, we reported all the empirical results obtained from FM-OLS technique. The estimated models (FM-OLS) support the hypothesized association of ICT export, ICT import, GDP per capita, economic complexity, and other control group variables with ecological footprints. The statistically significant and negative coefficient of ICT export (lnICT_exp_), research and development (lnRD), trade (lnTRA), and population (lnPOP) suggest that an upsurge in these factors reduce environmental degradation in terms of ecological footprint (lnEcoFP) in G-seven economies. On the other hand, the statistically significant and positive coefficients of ICT imports (lnICT_imp_), GDP per capita (lnGDPpc), and economic complexity (ECI) imply that an increase in these factors increase EcoFP in investigated regions. Precisely, the long-run responsiveness (elasticities) of ICT export, ICT import, GDP per capita, economic complexity, research and development, trade, and population are 0.091%, −0.079%, 0.193%, 0.071, −0.771%, −0.092%, and −2.044%, respectively (FM-OLS, column III). Furthermore, Table 6 reports more interesting empirical findings.

Frist, the coefficient of ICT export is positively and statistically significant with Eco-FP. On average, a 0.232% (FM-OLS I), 0.260% (FM-OLS II), and 0.091% (FM-OLS III) increase in EcoFP is caused by ICT export in the long run. It is worth mentioning that producing extra ICT-related heavy machines, equipment, and infrastructure and exporting of these goods are unfriendly to environmental quality, indicating that it is increasingly EcoFP. Therefore, our study suggests that ICT_export_ and its expansion might not be detected as a reasonable clarification in handling environmental issues [68]. These empirical results are in line with empirical work of [20] for 21 Sub-Saharan African countries, and [50] for G-seven economies. Our findings reveal that, although the production of ICT-related goods for exports has high production costs, overall, it contributes to environmental degradation. Particularly, it suggests that the governments and concerned policymakers of G-seven economies should further upgrade their machinery for the production of ICT-related goods to achieve high and sustainable economic growth and development. 

On the other hand, ICT import is statistically significant and negatively affects EcoFP in the long run. Precisely, a 0.162% (FM-OLS 1), 0.046% (FM-OLS II), and 0.079% (FM-OLS) decrease in EcoFP is caused by ICT import in the long run. These specific empirical results can be defendable by understanding the contributing role of the SE that is positively associated with environmental sustainability in terms of dematerialization, demobilization, and decarbonization activities. The substantial improvement in the ICT sector, especially in ICT import, encourages the implicit activities, for example, online business and academic conferences, online businesses, electronic books, e-commerce, and e-banking, decreases the aggregate demand for nonrenewable energy, and, therefore, decreases environmental unsustainability. The enhancement of SE in mitigating EcoFP has previously ignored the effect of consumption and cost effect. The empirical findings of this study are in line with the work of many researchers, who suggest that the ICT-import-based SE contributes to reducing emission levels by reducing the aggregate demand for energy consumption, encouraging resource competence, and technological development in the ICT sector [22,69]. The empirical results of all the panel models (FM-OLS I, II, and III) demonstrate the existence of big inconsistency between ICT_export_ and ICT_import_ (positive and negative) in the long run. Therefore, all countries from the G-seven region should plan and formulate their policies related to ICT according to the prevailing domestic economic situation. In this regard, the G-seven region considerately needs to increase private–public awareness about environmental issues by utilizing extra resources in their ICT sectors.

Regarding economic complexity (ECI), a 0.271% (FM-OLS I), 0.273% (FM-OLS), and 0.071% increase in EcoFP is caused by ECI in G-seven economies for the long run. These empirical results of ECI are in line with work of Khan et al. (2022) for the G-seven region, Neagu and Teodoru [24] for EU economies, and Pata [60] for USA. According to them, ECI has positively affected the production of greenhouse gas emission, representing risk-interfering emissions, in which export product’s complexity adds to rising EcoFP. Therefore, to construct complex products’ exports, these economies work hard to produce premium and heavy products with misuse of environmental resources. Consequently, the prevailing technological system and high aggregate demand of nonrenewable energy consumption degrades environmental quality in the investigated region.

When it comes to economic growth, the results suggest that economic growth (lnGDPpc) significantly contributes to environmental degradation, with a 0.130% (FM-OLS I), 0.132% (FM-OLS II), and 0.193% (FM-OLS III) increase in EcoFP caused by GDPpc growth or economic growth. The coefficients of GDPpc are statistically significant in all models (FM-OLS I, II, and III), as shown in Table 6. Likewise, GDP per capita is a best proxy for measuring economic growth of any country because it adds up many components of the economy, such as consumptions (C)+ investment (I)+ government expenditures (G)+, and net export (X-M). To the best of the authors’ knowledge, once the level of income upsurges in many developed and developing countries, it is most likely that not only individuals (households) and firms, but also government will increase spending (consuming), causing EcoFP to increase and, therefore, environmental pollution [5].

Expenditures on research and development (RD) suggest a significantly negative effect on EcoFP in G-seven economies for the long run. In Table 6, on average, a 0.670% (FM-OLS I), 0.779% (FM-OLS II), and 0.771% (FM-OLS III) decrease in EcoFP is caused by expenditure on RD. Particularly, research and development expenditure related to environment supports policymakers and government of these countries to recognize environmental influencing factors that are unfriendly to the environment. In addition, expenditures on RD can also help to find other substitutes that can be utilized to control the increasing demand of energy-intensive products. Furthermore, the government spending on RD helps to classify the tools and machineries that the industries can install as substitutes to energy-intensive tools and machineries and use environmentally friendly energies to improve environmental sustainability (Safi et al., 2021). These empirical results are in line with work of Fernández, et al. [70] for EU, USA, and China; Nguyen, Yandi and Mahaputra [34] for 13 G-20 economies; and Zhang, et al. [71] for Chinese industries.

In the case of control variables, foreign direct investment (FDI), and all models (FM-OLS I, II, and III) of Table 6, there is a statistically insignificant relationship between FDI and EcoFP, even at the 10% level in the long run. These empirical results are consistent with empirical work of [72] for Pakistan. Likewise, the coefficient of trade ratio (lnTRA) is statistically insignificant in model 2 (FM-OLS II), while it became significant in model 3 (FM-OLS III) with a negative sign, indicating that a 0.092% decrease in EcoFP was caused by 1% increase in trade. Similarly, population has a direct association with EcoFP. More precisely, a 1% increase in population will lead to increase in EcoFP by 2.044% in G-seven economies for the long-run equilibrium. These findings are in line with empirical work of Khan and Yahong [72] for Pakistan; Khan and Yahong [35]; and Khan, Yahong and Zeeshan [1] for 18 Asian emerging and less developed economies. It is also worth mentioning that this study applied the PMG-ARDL econometric technique to verify the validity and accuracy of our main models (FM-OLS I, II, and III) and check the predictability of all variables used in regression models. The empirical findings in Table 7, obtained from PMG-ARDL (PMG I, II, and III) are in line with the findings of FM-OLS models, which verify the robustness of our results. PMG-ARDL methodology consider cross-sectional dependency between different cross-sections (here, countries) and heterogeneity through the parameter estimates for the short run. In addition, this econometric technique also enables the long-run causality inferences and short-run causality inference, which can be drawn irrespective of whether the series (variables) are integrated with first difference or at level. In the existing environmental economics literature, Mensah, Long, Boamah, Bediako, Dauda and Salman [54] for African countries; and Banday and Aneja [73] and Raheem, Tiwari and Balsalobre-Lorente [50] for G-seven countries used the similar application of PMG-ARDL.

## 5. Conclusions

In this study, we critically analyzed existing papers in the environmental economics literature, in which many researchers used carbon emissions as a proxy for environmental unsustainability, which cannot reflect the major parts of the ecosystem. By taking this into account, in this study, we analyze the linkages between information, communications and technology, economic complexity, and ecological footprint (along with other control variables, such as economic growth, FDI, research and development, trade ratio, and population in G-seven countries. The G-seven countries (Canada, France, Germany, Italy, Japan, UK, and USA) are producing more emissions, which put pressure on the environmental quality. Using different econometric methods, such as panel unit root/stationarity tests (both first and second-generation), panel cointegration tests (Pedroni and Kao), and panel regression analysis (FM-OLS and PMG-ARDL), the empirical evidence obtained from this study concludes that ICT export increases the EcoFP in the long run for G-seven economies, while ICT import contributes to reducing EcoFP in these regions. Furthermore, the empirical findings conclude that economic complexity increases environmental sustainability and plays a more detrimental role to increase EcoFP in all cross-sections. However, the elasticities of coefficients are divers for all regression models used in this study. In addition, economic growth or per capita GDP also play a harmful role by reducing environmental sustainability, whereas FDI does not contribute to affecting EcoFP in the long run. As expected, research and development significantly reduce ecological destructions by spending on environmental-related research and development programs. On the other hand, trade plays a vital role to reduce environmental pollution in the long run, whereas population played a very destructive role to degrade environmental quality in the G-seven region.

The specific policy implications and discoveries can be summarized from the empirical findings of this study as follows:

I. ICT export and ICT import positively and negatively influence environmental sustainability in terms of ecological footprint, respectively. This empirical finding implies that importing highly advanced ICT infrastructure can help to decrease environmental unsustainability in the investigated region. Meanwhile, the production and export of these (ICT infrastructure) might be paid as high environmental cost. This evidence suggests that the governments and policymakers of these regions should implement policy to encourage the penetration of the ICT sector to maintain environmental sustainability.
II. The significance of ECI in boosting the economy has been widely considered and accepted in this role. However, current debates on controlling effects of ECI to environmental aspects also draw enormous attention from a wide range of policymakers and concerned authorities. By the same token, the empirical results for economic complexity positively affect EcoFP. This infers that prevailing economic transformation (to knowledge- and skill-based) of industrial structure and economic activities in selected G-seven economies exploits environmental sustainability and is not environmentally friendly. Therefore, the concerned authorities should consider economic activities and complex structure of industries while implementing environmental sustainability policies.
III. Likewise, the empirical evidence provides that FDI and trade activities will not help G-seven economies to reduce environmental unsustainability by lowering ecological footprint. Foreign investors and trade are not helpful to bringing environmentally friendly technology to the host countries to reduce environmental unsustainability. Therefore, the governments of the investigated region should encourage foreign investors and domestic traders to bring greener technology, which will not only help the G-seven countries in environmental terms, but also in economic condition.
IV. Regarding research and development, it also negatively affects environmental unsustainability by reducing ecological footprint. The governments of the investigated region should invest more in research and development to reduce the harmful effect of economic growth on the environment. The research and development can also be helpful in structure transformation of the economy toward green economy.

In order to strengthen the existing qualitative and quantitative information, the research gap can be highlighted in light of the following caveats and limitations. First, the current study’s emphasis is on the relationship between ICT, ECI, and EcoFP; therefore, it would be of more robust and interesting if future researchers inspect the relationship between ICT and EcoFP on a micro or macro level (firms or industrial level), specifically, analyzing the relationship between ICT and environmental sustainability in different firms and industries in the G-seven countries. Second, another econometric method (alternative methodologies) should be used to estimate whether the empirical findings of this study are in line with the empirical examination within the homogenous (standardized) panel and country-specific analysis.

The current study also leaves the gap for future researchers to fill by investigating developing or emerging economies. The investigation of the current study considered only developed economies; therefore, the findings for developing or emerging economy regions from Asia or sub-Saharan Africa must be different. Thus, future research would improve policy effect and explore many research objectives not even in developed but in the developing world. Moreover, in the analysis of this study, we could not find the short-run elasticities and causality among the variables because of inaccessibility to an extended dataset. It would be a more valid and reliable outcome to handle this constraint in future research. Lastly, this study could not perform the causality and empirical mechanism tests between variables used in the models (due to unavailability of a longer dataset for longer panel). Therefore, it would be better to cover this gap in the future study by conducting empirical mechanism tests to verify the impact mechanism and causality between variables.

## Figures and Tables

**Table 1:** Acronym, measuring unit, nature, and source of the variables.

Variables	Abbreviations	Measures	Nature	Source
Ecological footprint	EcoFP	Per capita ecological footprint (in global hectares)	Main	Global Footprint Network (GFN)
Informational communication and technology (export)	ICT_exp_	Export of ICT-related goods	Main	World Development Indicators (WDI) https://databank.worldbank.org/ (accessed on 23 February 2022).
Informational communication and technology (import)	ICT_imp_	Import of ICT-related goods	Main	WDI
Economic Complexity (index)	ECI	The production composition face of a country by installing and including the information of their variety of range (the number of exported product)	Main	ALTAS of Economic Complexity https://atlas.cid.harvard.edu/rankings (accessed on 23 February 2022).
Economic growth	GDPpc	Gross domestic products per person (measure in current price of USD)	Main	WDI
Foreign Direct Investment	FDI	Net inflow of FDI (percentage of GDP)	Control	WDI
Research and Development	RD	Expenditure on research and development (percentage of GDP)	Control	WDI
Trade Ratio	TRA	Trade ratio in percentage of GDP	Control	WDI
Population	POP	People living per square kilometer of land area (population density)	Control	WDI

**Table 2:** Summary statistics of the variables.

Variables	lnEcoFP	lnICT_exp_	lnICT_imp_	lnGDPpc	lnECI	lnFDI	lnPOP	lnTRA	lnRD
Mean	1.777	1.713	2.207	10.580	0.439	0.296	4.541	3.892	0.739
Max	2.324	3.013	2.749	11.052	1.022	2.478	5.861	4.482	1.223
Min	1.430	0.512	1.569	9.927	−0.616	−6.393	1.241	2.973	0.039
S.D.	0.261	0.671	0.318	0.213	0.390	1.236	1.500	0.393	0.325
Skew	0.691	0.100	−0.093	−0.726	−1.089	−2.130	−1.274	−0.689	−0.422
Kurt	2.109	2.111	2.032	3.693	3.814	10.796	3.240	2.338	2.198
JB	14.20	4.353	5.100	13.600	28.419	397.97	34.39	12.292	7.061
Prob	0.000	0.113	0.078	0.001	0.000	0.000	0.000	0.002	0.029

Note: the calculations are based on log values, the average value of the variable is denoted by Mean, Max is maximum value, Min is minimum, S.D. represents standard deviation of variables, Skew and Kurt indicates skewness and kurtosis in variables, JB and Prob represents Jarque & Bera tests and its probability values.

**Table 3:** Cross-sectional dependence.

Variables	Pesaran CD	B-P LM	H0
lnEcoFP	15.41 ***	242.94 ***	Rejected
lnICT_exp_	18.92 ***	325.50 ***	Rejected
lnICT_imp_	14.05 ***	205.97 ***	Rejected
lnGDPpc	14.88 ***	233.47 ***	Rejected
lnECI	12.44 ***	177.70 ***	Rejected
lnFDI	2.20 **	28.49	Rejected at CD
lnPOP	4.85 ***	208.22 ***	Rejected
lnTRA	8.08 ***	209.89 ***	Rejected
lnRD	5.09 ***	202.06 ***	Rejected

** (2 asterisks) denotes 5% and *** (3 asterisks) denotes 1% level of significance.

**Table 4:** Panel unit root tests.

Variable	Level		First Difference		Order
	No Trend	with Trend	No Trend	with Trend	
IPS (2003)					
lnEcoFP	2.8165	−0.4742	−4.0519 ***	−3.0111 ***	1st difference
lnICT_exp_	0.4289	2.1736	−3.2769 ***	−2.9242 ***	1st difference
lnICT_imp_	−1.1060	−0.7257	−5.7259 ***	−4.4426 ***	1st difference
lnGDPpc	−3.9456 ***	−1.8660 **	-	-	Level
lnECI	−1.3804 *	1.1144	−4.2139 ***	−3.5384 ***	1st difference
lnFDI	−2.4363 ***	−2.3832 ***	-	-	Level
lnPOP	0.0863	2.5328	0.1925	−1.3963 **	1st difference
lnTRA	−0.6490	−0.9442	−4.8942	−3.8293	1st difference
lnINF	−2.3324	−1.7257	−3.0346 ***	−2.8874 ***	1st difference
lnRD	2.1801	−1.4298 *	−3.2647 ***	−1.4815 **	1st difference
Cross-sectional IPS (CIPS)					
lnEcoFP	−2.361 **	−2.129	-	-	Level
lnICT_exp_	−1.934	−2.254	−3.886 ***	−3.950 ***	1st difference
lnICT_imp_	−2.365 **	−2.744	-	-	Level
lnGDPpc	−1.399	−1.998	−3.101 ***	−3.028 ***	1st difference
lnECI	−2.721 ***	−4.269	-	-	Level
lnFDI	−3.232 ***	−3.292 **	-	-	Level
lnPOP	−0.867	−0.987	−3.077**	−1.357	1st difference
lnTRA	−1.248	−1.612	−2.483 **	−2.942 ***	1st difference
lnRD	−2.160	−2.359	−4.133 ***	−4.141 ***	1st difference

* (1 asterisk) denotes 10%, ** (2 asterisks) denotes 5%, and *** (3 asterisks) denotes 1% level of significance.

**Table 5:** Cointegration tests.

Pedroni			Kao	
Dimensions			ADF	
Statistics	Within-Dim	Between-Dim	t-Statistics	Prob.
Panel v-stat	1.691 **	-	−3.7063 ***	0.0001
Panel PP-stat	−2.616 ***	-	-	-
Panel ADF-stat	−2.758 ***	-	-	-
Group PP-stat	-	−4.885 ***	-	-
Group ADF-stat	-	−3.858 ***	-	-

** (2 asterisks) denotes 5%, and *** (3 asterisks) denotes 1% level of significance.

**Table 6:** Estimations under the FM-OLS methodology.

Variables	FM-OLS I	FM-OLS II	FM-OLS III
lnICT_Exp_	0.232 *** (0.024)	0.260 *** (0.021)	0.091 *** (0.011)
lnICT_Imp_	−0.162 *** (0.046)	−0.172 *** (0.038)	−0.079 *** (0.018)
lnGDPpc	0.130 *** (0.035)	0.132 *** (0.029)	0.193 *** (0.013)
lnECI	0.271 *** (0.069)	0.273 *** (0.057)	0.071 *** (0.027)
lnFDI	−0.002 (0.004)	−0.005 (0.003)	−0.001 (0.002)
lnRD	−0.670 *** (0.073)	−0.779 *** (0.069)	−0.771 *** (0.031)
lnTRA	-	0.160 *** (0.042)	−0.092 *** (0.010)
lnPOP	-	-	2.044 *** (0.076)
Models’ statistics			
R2	0.955199	0.956982	0.979361
Adjusted-R2	0.949599	0.951095	0.976287

*** (3 asterisks) denotes 1% level of significance (the values in parenthesis represent standard error of the coefficient).

**Table 7:** Estimations under PMG-ADRL methodology.

Variables	PMG I	PMG II	PMG III
lnICT_Exp_	0.582 ***(0.071)	0.694 *** (0.043)	0.689 *** (0.055)
lnICT_Imp_	−0.250 ***(0.105)	−0.475 *** (0.086)	−0.375 *** (0.088)
lnGDPpc	0.166 ***(0.025)	0.227 *** (0.030)	0.237 *** (0.050)
lnECI	0.416 ***(0.128)	0.461 *** (0.111)	0.221 *** (0.064)
lnFDI	0.081 * (0.043)	0.050 ** (0.001)	0.018 (0.002)
lnRD	−0.177 *** (0.078)	−0.011 *** (0.044)	−0.588 *** (0.065)
lnTRA	-	0.068 (0.058)	0.188 *** (0.028)
lnPOP	-	-	2.210 *** (0.153)
Models’ statistics			
Dep: lags	1 (fixed)	1 (fixed)	1 (fixed)

* (1 asterisk) denotes 10%, ** (2 asterisks) denotes 5%, and *** (3 asterisks) denotes 1% level of significance (the values in parenthesis represent standard error of the coefficient).

## Data Availability

The material and data that support the empirical findings of this study are accessible at: https://datacatalog.worldbank.org/dataset/world-developmentindicators (accessed on 23 February 2022); https://www.footprintnetwork.org/our-work/ecological-footprint/ (accessed on 23 February 2022); https://atlas.cid.harvard.edu/rankings (accessed on 23 February 2022).

## Appendix A

**Table A1:** Literature review’s summary in tabulated form.

Authors	Regions	Time Periods	Methodology	Results
Relationship between ICT and environmental unsustainability				
[13]	Caribbean and Latin American economies	1995 to 2017	Continuously upgraded and fully modified (CUP-FM) and Biased corrected (CUP-BC) models	The results obtained from the models suggest that ICT increases environmental sustainability by reducing carbon emission.
[12]	Six ASEAN economies	1995 to 2017	Generalized method of moment (GMM), CUP-FM and CUP-BC models	ICT decreases the intensity of carbon emissions in ASEAN economies
[40]	Tunisian economy	1975 to 2014	Autoregressive Distributed Lag (ARDL)	The suggested empirical evidence indicates statistically insignificant relationship between ICT and carbon emission in investigated region.
[74]	Forty-four Sub-Sharan African economies	2000 to 2012	GMM	There is negative relationship between ICT and carbon emissions in targeted region.
[20]	Twenty-one Sub-Saharan African economies	1996 to 2014	Panel Corrected Standard Error (PSCE) and Feasible Generalized Leas Square (FGLS)	ICT, energy consumptions, and carbon emissions have positive relationship in Sub-Saharan African countries
[15]	Chinese provinces	2001 to 2016	Panel quantile regression model (PQR)	All measures of ICT support to decrease carbon emissions in Chinese economy
[17]	European Union (EU)	2001 to 2014	ARDL-Pooled Mean Group (ARDL-PMG)	The use of internet (proxy for ICT) declines environmental sustainability. The findings also suggest that EU economies did not achieve the goal of green economy.
[19]	Iran	2002 to 2013	Dynamic OLS	The findings suggest different results, such as ICT in services and transportation sector have negative effect on environmental degradation while, in the industrial sector, ICT and carbon emissions have positive relationship
[22]	BRICS countries	1990 to 2015	CUP-BC and CUP-FM	The development of ICT significantly reduces carbon emissions in investigated region
[61]	Nine Asian countries	1990 to 2018	ARDL	The emergence of ICT in economy significantly influences carbon emissions in all countries.
Relationship between ECI and environmental unsustainability				
[75]	Portugal, Ireland, Italy, Greece, and Spain (PIIGS) economies	1990 to 2019	Dynamic OLS	The empirical findings suggest the nonlinear relationship between ECI and carbon emission. This relationship supports EKC hypothesis and N-shaped relationships in the long run.
[76]	France	1964 to 2014	Dynamic OLS	The results show that ECI overturns the intensity of carbon emissions in France. The empirical evidence also supports the existence of U-shaped association between variables
[57]	One hundred and eighteen developed and developing economies	2002 to 2014	System-GMM	ECI has significantly positive effect on carbon emissions in investigated region
[56]	Twenty-eight OECD economies	1990 to 2014	Augmented Mean Group (AMG), Common correlated AMG (CCEMG), ARDL, Dynamic OLS, and FM-OLS	ECI reduces environmental unsustainability and can help to mitigate environmental effects
[5]	G-seven economies	1996 to 2019	Fully modified ordinary least square (FM-OLS) and Dynamic OLS (DOLS)	The linear impact of ECI declines environmental sustainability, while the nonlinear (ECI2) supports the presence of EKC hypothesis
[24]	EU	1995 to 2016	FM-OLS and DOLS	ECI significantly affects greenhouses green emissions in EU economies for the long run.
[9]	United States of America	1965 (Quarter-1) to 2017 (Quarter-4)	Quantile autoregression distributed lag (QARDL) model	ECI significantly increases EcoFP in USA.
